# Effect of Diffusion on Discoloration of Congo Red by Alginate Entrapped Turnip (*Brassica rapa*) Peroxidase

**DOI:** 10.1155/2015/575618

**Published:** 2015-02-05

**Authors:** Afaf Ahmedi, Mahmoud Abouseoud, Amrane Abdeltif, Couvert Annabelle

**Affiliations:** ^1^Laboratoire de Biomatéraiux et Phénomènes de Transport, Faculté des Sciences et de la Technologie, Université de Médéa, Pole Universitaire, RN1, 26000 Médéa, Algeria; ^2^Laboratoire de Génie de la Réaction, Faculté de Génie Mécanique et Génie des Procédés, Université Houari Boumediene, 16111 Bab Ezzouar, Algeria; ^3^Ecole Nationale Supérieure de Chimie de Rennes, CNRS, UMR 6226, avenue du Général Leclerc, CS 50837, 35708 Rennes Cedex 7, France; ^4^Université européenne de Bretagne, 35000 Rennes, France

## Abstract

Enzymatic discoloration of the diazo dye, Congo red (CR), by immobilized plant peroxidase from turnip “*Brassica rapa*” is investigated. Partially purified turnip peroxidase (TP) was immobilized by entrapment in spherical particles of calcium alginate and was assayed for the discoloration of aqueous CR solution. Experimental data revealed that pH, reaction time, temperature, colorant, and H_2_O_2_ concentration play a significant role in dye degradation. Maximum CR removal was found at pH 2.0, constant temperature of 40°C in the presence of 10 mM H_2_O_2_, and 180 mg/L of CR. More than 94% of CR was removed by alginate immobilized TP after 1 h of incubation in a batch process under optimal conditions. About 74% removal efficiency was retained after four recycles. Diffusional limitations in alginate beads such as effectiveness factor *η*, Thiele modulus Φ, and effective diffusion coefficients (*D*_*e*_) of Congo red were predicted assuming a first-order biodegradation kinetic. Results showed that intraparticle diffusion resistance has a significant effect on the CR biodegradation rate.

## 1. Introduction

Approximately 10000 different dyes and pigments are produced annually worldwide and used extensively within food, pharmaceutical, cosmetic, printing, textile, and leather industries. It is estimated that between 10 and 20% of about 7 × 10^5^ tons of dyestuff being manufactured each year and used in dyeing processes may be found in wastewater [1]. Azo dyes are the major group of dyestuffs and have been identified as the most problematic compounds in textile effluents due to their higher water solubility and lower degradability [2]. Several of these dyes are very stable to light, temperature, and microbial attack and contribute to organic load and toxicity of the wastewater [3]. Furthermore, they pose a problem because of their carcinogenicity and toxicity. Therefore, removal of such dyes before discharging them into natural water streams is essential. Thus, appropriate treatment technologies are required. The treatment of recalcitrant and toxic dyes with traditional technologies is not always efficiently done or may not be environmentally friendly [4].

Several physicochemical treatment methods for the removal of color from industrial wastewaters such as ozonation, Fenton reaction [5, 6], and adsorption [7, 8] were applied. However, these technologies are usually costly and are not easily adapted [9]. The use of bacteria in the biological treatment of dye effluents may result in the generation of colourless, dead-end aromatic amines which are generally more toxic than the parent compounds and thus have poor adaptability and limited application to a wide range of dye wastewaters [10, 11].

Oxidoreductive enzymes such as peroxidases and polyphenol oxidases are participating in the degradation and removal of aromatic pollutants from various contaminated sites [12]. These enzymes can act on a broad range of substrates and can also catalyze the degradation and facilitate the removal of organic pollutants present at very low concentrations in the contaminated site or in wastewaters [13]. In view of the potential of these enzymes in treating the phenolic compounds several microbial and plant peroxidases and polyphenol oxidases have been considered for the treatment of dyes [14, 15]. Specifically, the use of plant peroxidases in removal of phenolic pollutants from aqueous solution is well documented. Turnip roots, which are readily grown in several countries, are a good source of peroxidase and because of their kinetic and biochemical properties they have a high potential as an economic alternative to HRP [16, 17].

However, the use of soluble enzymes has some inherent limitations as compared to immobilized form of enzymes [18, 19], which has several advantages over the soluble enzymes such as enhanced stability, easier product recovery and purification, protection of enzymes against denaturants, and reduced susceptibility to contamination and offer the possibility of reutilizing the enzyme [20]. Entrapment in alginate beads is also known to be a simple, nontoxic, lower cost method [21].

Several methods have been compared and reported for the immobilization of peroxidase on different supports which provide useful information on the efficiency of the degradation of azo dyes [22, 23]. The catalytic activity of the immobilized enzyme is affected mainly by the limitations of internal and external mass transfer [24, 25]. Enzyme biochemical properties and reaction type and kinetics as well as support chemical and mechanical properties all affect the internal mass transfer [26]. The estimation of the dimensionless parameters *η* and Φ, known, respectively, as effectiveness factor and Thiele modulus, is very important because they relate the reaction rate to diffusion rate [27]. On the other hand, knowledge of the effective diffusion coefficients, *D*_*e*_, of reacting compounds is of crucial importance for the quantitative analysis of bioprocesses using immobilized biocatalysts [28].

This work is an extension of our previous research done in the laboratory using partially purified enzyme extracted from turnip “*Brassica rapa”* for the discoloration of an azo dye. In a previous study, the effects of various operating conditions on dye discoloration were analyzed aiming to reach maximal colour removal. In present study, the use of immobilized TP in batch discoloration of a model azo dye, Congo red (CR), is discussed using calcium alginate gel beads as the support material. Several important factors such as pH, contact time, temperature, dye, and hydrogen peroxide concentration which may affect dye biodegradability by immobilized turnip “*Brassica rapa” *peroxidase by entrapment on calcium alginate are also investigated and reported in order to select optimal operating conditions for maximal discoloration. Results could be compared to those obtained in an earlier work with free TP [29]. The key parameters giving an estimation of internal mass-transport resistance in alginate particles were quantified and discussed.

## 2. Theory

Diffusional phenomena play an important role in heterogeneous enzymatic processes. External diffusion is often negligible due to efficient mixing of the solution. Thus, although more complex, the internal diffusional limitations have received important theoretical attention, in particular when coupled to chemical reaction [30, 31].

Some parameters could be calculated in order to quantify the contribution of mass transfer by diffusion into support particles on the overall transformation process rate. Diffusional limitations can be quantitatively expressed by the effectiveness factor, *η*, defined as the ratio between the average reaction rate and the rate that would be obtained if all enzyme molecules inside the particles were exposed to the same substrate concentration as the bulk liquid, that is, in the absence of diffusional effects. Thus, the relative influence of diffusion on biochemical reaction rate can be expressed by the following equation [32, 33]:

(1) $$\begin{array}{c} \eta =\frac{\text{actual}\;\text{apparent}\;\text{rate}}{\text{rate}\;\text{at}\;\text{bulk}\;\text{liquid}\;\text{concentration}}. \end{array}$$

The effectiveness factor *η* is a dimensionless parameter that measures how effectively the catalyst is being used. For *η* near unity, the entire volume of the particle is reacting at the same high rate because the reactant is able to diffuse quickly through the support material. For *η* near zero, the reaction is conducted at the lowest rate. The reactant is unable to penetrate significantly and the reaction rate is limited in a small portion of the particle volume. The diffusional resistance is predominant which lowers the overall reaction rate [34].

Assuming that enzyme molecules are uniformly distributed in a spherical support particle and there is no partitioning of the substrate between the exterior and the interior of the support, the following equation can be written on the basis of Fick's law stating that diffusion rate is equal to reaction rate at steady state [35]:

(2) $$\begin{array}{c} \frac{v_{r}}{D_{e}}=\frac{d^{2}C}{{dr}^{2}}+\frac{2}{r}\frac{dC}{dr}. \end{array}$$

Assuming that the biodegradation kinetics is expressed by first order kinetics (which is a correct assumption, especially at lower substrate concentrations), the relation between biodegradation rate and substrate concentration is given as

(3) $$\begin{array}{c} v_{r}=kC, \end{array}$$

where *v*_*r*_ = actual biodegradation rate of Congo red ((mg/L of dye)/min) at radius *r*; *C* = azo dye concentration at radius *r* within the bead (mg/L); *k* = the first-order biodegradation rate constant (min^−1^); *D*_*e*_ = effective diffusion of substrate coefficient in the particle, and *r* = radial coordinate.

The overall effectiveness factor includes the effects of intraparticle diffusional resistance. For an irreversible first order reaction; (2) becomes

(4) $$\begin{array}{c} \frac{kC}{D_{e}}=\frac{d^{2}C}{{dr}^{2}}+\frac{2}{r}\frac{dC}{dr}. \end{array}$$

Solution of (4) to give a concentration profile allows *η* to be evaluated. The result is

(5) $$\begin{array}{c} \Phi =\frac{1}{\eta }\left(\frac{1}{\tanh3\Phi }-\frac{1}{3\Phi }\right). \end{array}$$

The* Thiele modulus* is the ratio of the intrinsic chemical reaction rate in the absence of mass transfer limitation to the rate of diffusion through the particle. For large values of the Thiele modulus, the rate of reaction is much greater than the rate of diffusion, the effectiveness factor is much less than unity and *η* ≈ 1/Φ, and we say the pellet is* diffusion limited*. Conversely, at small Φ when the diffusion rate is much larger than the reaction rate, the effectiveness factor is near unity, and we say the system is* reaction limited* [34].

Inside the catalyst particles, diffusion of the substrate takes place in the cavities of the catalyst pores filled with liquid. This complex interaction is modeled by assuming an overall diffusion process, determined by a coefficient *D*_*e*_, called effective diffusion coefficient, which takes into account the porous geometry of the support [36, 37]. The value of *D*_*e*_ can be determined experimentally and is a property of the solute and the solvent [38]. Effective diffusion *D*_*e*_ can be calculated with this formula:

(6) $$\begin{array}{c} D_{e}=\frac{R^{2}}{9}\frac{k}{\Phi^{2}}, \end{array}$$

where *R* is the radius of immobilized particle (cm). This formula was applied to estimate the *D*_*e*_ of Congo red in the calcium alginate gel with immobilized TP.

## 3. Material and Methods

### 3.1. Dye

Congo red (CR), acetone, hydrogen peroxide (H_2_O_2_), and 4-aminoantipyrine were all purchased from Sigma-Aldrich (St. Louis, MO, USA). Detailed properties of the dye along with the structure are presented in Table 1.

### 3.2. Extraction and Partial Purification of TP

Turnip roots were collected from local market. Turnip (50 g) was homogenized in 100 mL of distilled water. Homogenate was filtered through four layers of cheesecloth. The filtrate thus obtained was subject to solvent fractionation by adding acetone. The crude enzyme was treated with three volumes of cold acetone (4°C) and left for 3 hours under constant agitation in an iced bath to obtain maximum TP precipitate. The precipitate was collected by centrifugation at 4000 g on a Remi R-24 cooling centrifuge. The precipitated enzyme was collected and dissolved in sodium phosphate buffer 10 mM, pH 6.0.

### 3.3. Immobilization of TP by Entrapment in Calcium Alginate Gel Beads

The purified enzyme was used for immobilization into alginate (PANREAC QUIMICA SA, (Bercelona) ESPANA). The immobilization procedure was similar to that described in several works [21, 41]. A volume of 10 mL of semipurified TP solution (containing 1.25 U·mL^−1^) was thoroughly mixed with 1.5% of sodium alginate and was kept under agitation until a homogenous solution was obtained. The resulting mixture of alginate and enzyme was dropped through a syringe needle to form small droplets into an agitated CaCl_2_ solution (0.05 M). Alginate calcium beads of approximately 3.2 mm diameter were instantaneously formed. The agitation was maintained for about 2 hours in order to harden and stabilize the enzyme in alginate beads. Finally, beads were washed with distilled water and conserved at 4°C in a buffer solution pH 6.0 before further use.

### 3.4. Enzyme Assay

TP activity was assessed by the 4-aminoantipyrine method using phenol and H_2_O_2_ as substrates and 4-aminoantipyrine as a chromogen [42]. The rate of H_2_O_2_ consumption in the assay was calculated from the rate of formation of the colored product with a *λ*_max⁡_ at 517 nm and a molar absorptivity of 5680 mol^−1^ L cm^−1^.

One unit of activity (U) is defined as *μ*mol H_2_O_2_ consumed per min.

Specific activity (activity/protein concentration) was estimated by assaying protein concentration by the standard Bradford method [43].

### 3.5. Quantitative Estimation of Dye Concentration

Quantitative estimation of the Congo red azo dye in the aqueous phase was carried out by a colorimetric method using a spectrophotometer in the visible range. A solution of 40 mg/L concentration was scanned over a range of 190–800 nm by using UV-vis spectrophotometer and *λ*_max⁡_ wavelength was determined to be 500 nm. A standard curve was prepared at maximum wavelength  *λ*_max⁡_ and was used for the estimation of the dye concentration in aqueous phase. After treatment, the sample was centrifuged and the supernatant was assayed for the residual dye concentration [44].

### 3.6. Removal of Congo Red Azo Dye in Aqueous Phase by Immobilized Enzyme

Experiments were conducted by using one-factor-at-a-time (OFAT) method [45]. The studied parameters were pH (2–10), reaction time (0–60 minutes), temperature (20–70°C), CR concentration (20–200 mg/L), and H_2_O_2_ concentration (0.01–1 M). Initial assays were carried out in a series of stirred vials containing 50 mg/L dye, 0.7 g of immobilized TP, and H_2_O_2_ dose of 50 mM. After one hour, the treated solution was centrifuged at 4000 rpm for 5 min in order to separate the precipitate. Residual dye concentration was estimated spectrophotometrically at 500 nm. Optimum values where those giving the highest discoloration yield. All experiments were done in triplicate. Control (blank) experiments were performed in the absence of immobilized TP to assess abiotic (nonenzymatic) degradation via photocatalytic or chemical processes.

Discoloration yield was calculated for Congo red dye. Parameter percent decolorization was defined as

(7) $$\begin{array}{c} \text{Dye}\;\text{removal}\left(\%\right)=\frac{A_{0}-A_{t}}{A_{0}}\times 100, \end{array}$$

where *A*_0_ and *A*_*t*_ are the absorbance before and after enzymatic treatment, respectively.

### 3.7. Reusability of Immobilized TP

The reusability of immobilized TP was studied by repeated use of beads for dye removal under fixed optimal conditions.

Experiments were performed repeatedly using the same sample of immobilized TP (approximately 0.7 g) in ten consecutive agitated batches Once reaction was completed, beads containing immobilized TP were separated from the mixture, washed with distilled water, and used again in a fresh decolorization medium. Dye decolorization was monitored by UV-vis at *λ*_max⁡_ (500 nm) at the end of each batch.

## 4. Results and Discussion

### 4.1. Extraction, Purification, and Immobilization of Peroxidase

The abundance and the simplicity of extraction of plant peroxidase are amongst many reasons of their choice as a source of oxidoreductive enzymes [46]. The turnip “*Brassica rapa*” presents the highest peroxidase activity compared to other sources of vegetables [29]. The semipurified enzyme was less denatured and was more stable than the raw enzyme.

The experiments were designed to assess the dye discoloration in the presence of H_2_O_2_ and crude TP; interestingly it proved to be a very good enzyme. The dye precipitation was a result of H_2_O_2_-dependent enzymatic reaction, possibly involving free-radical formation followed by polymerization and precipitation.

The objective of the present study is to obtain the maximum degradation percentage of Congo red by immobilized TP on calcium alginate with the minimum quantity of inputs, minimizing the process cost. A one factor at a time strategy was adopted to optimize different parameters (pH, contact time, temperature, dye concentration, and quantity of H_2_O_2_) that could affect the enzymatic degradation of CR.

We have first ever reported the application of partially purified TP in the discoloration of textile and other industrially important dyes. In order to reduce the cost of the wastewater treatment, simple acetone precipitated proteins from turnip were taken for the treatment of a Congo red present in polluted wastewater. Partially purified preparation of TP was obtained by adding acetone.

Results of TP purification protocols are summarized in Table 2. The enzyme was purified about 1.3 times with a recovery of 82.78%. The extraction conditions resulted in the enrichment of enzyme specific activity (6.34 U/mg), which is due to the differential partitioning of the desired enzyme and contaminating enzymes: proteins to the opposite phases. This specific activity is comparable to those obtained for plant or fungal peroxidase [47]. Better results could be obtained by a combination of conventional methods such as ammonium sulfate precipitation, acetone fractionation, and column chromatography [48]. Nevertheless, a higher cost could be recorded with no significant improvement in treatment efficiency.

### 4.2. Optimum pH

**Figure 1:**
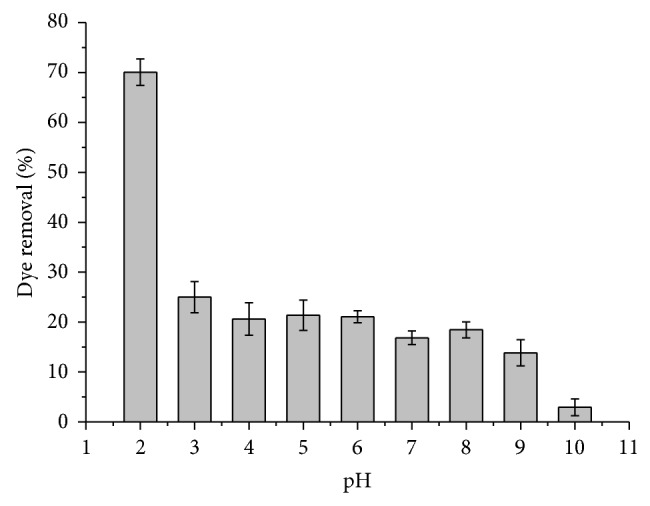
Effect of pH on the removal of 50 mg/L of Congo red azo dye in the presence of 0.7 g of immobilized TP and 50 mM of H_2_O_2 _for 1 h in the buffers of different pH. The molarity of each buffer was 10 mM.

Most enzymes have a characteristic pH value at which their activity is maximized. The studies were carried out on the Congo red azo dye by varying aqueous-phase pH of the reaction mixture between 2 and 10 at fixed dye and H_2_O_2_ concentrations, reaction temperature (25°C), and contact time. Variation of dye removal at various pH values is depicted in Figure 1.

The discoloration efficiency was maximum (70%) at pH 2.0 and then decreased with increasing pH (Figure 1). At neutral pH, CR removal did not exceed 18%. The interrelation of enzymatic activity with pH, for any enzyme, depends on the acidic–basic behavior of the substrate, as well as other factors which are, in general, difficult to analyze quantitatively. A similar result was obtained by other authors [13, 49].

### 4.3. Incubation Time

**Figure 2:**
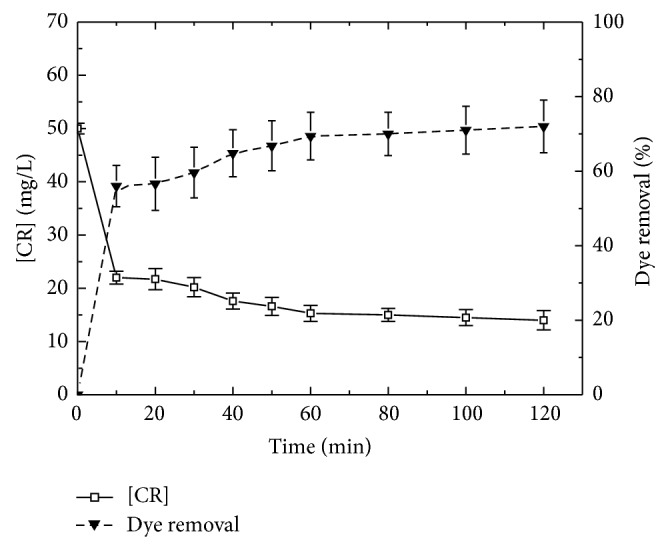
Effect of contact time on dye removal with immobilized TP. (50 mg/L of CR azo dye in the presence of 0.7 g of immobilized TP and 50 mM of H_2_O_2 _in buffer pH 2.0 at 24°C).

The effect of contact time on the discoloration efficiency of Congo red solution was analyzed under fixed substrate concentrations, temperature, and optimal pH 2. From Figure 2, 56% of colour was removed after only 10 minutes. Longer exposure slowed the discoloration process and a final yield of 72% was obtained after 2 hours.

The fact that the reaction became slower may be attributed to the simultaneous decrease of reacting substrates (dye and H_2_O_2_). We can conclude that the enzymatic reaction of TP is fairly rapid, with most of the color removed in the first 10 min of contact time. This duration is not a final choice as other parameters may contribute in rate and yield increase.

### 4.4. Temperature

**Figure 3:**
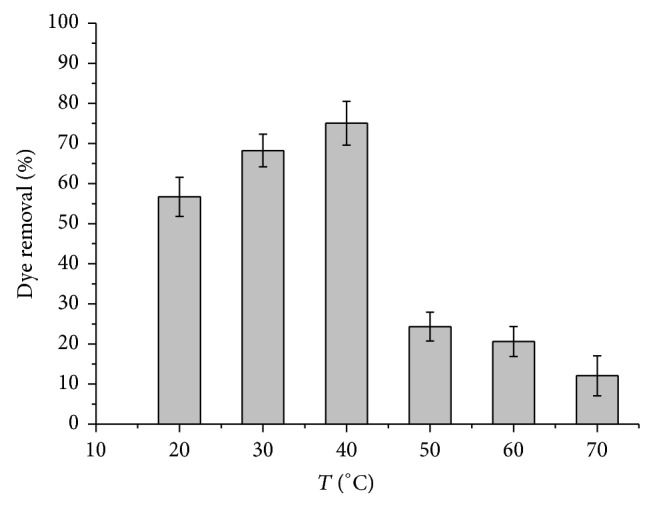
Effect of temperature on the discoloration of CR by immobilized TP. (50 mg/L CR, 50 mM of H_2_O_2_, pH 2.0 and 0.7 g of immobilized TP for 10 min).

The effect of temperature on the discoloration of CR by TP enzyme was shown in Figure 3. Maximum color removal of CR was observed between 30°C and 40°C with about 73% but temperature 40°C showed a slightly higher percentage color removal. The denaturation effect on immobilized TP was observed for higher temperatures. More than 70% discoloration efficiency was lost when temperature exceeded 40°C.

### 4.5. Optimum Dye Concentration

**Figure 4:**
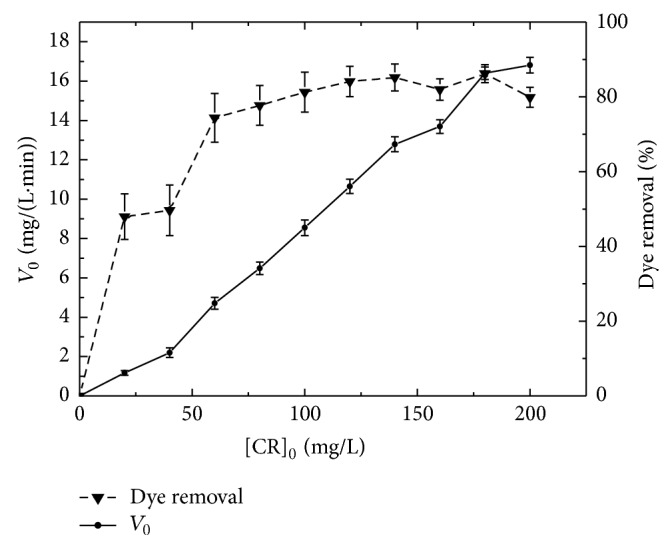
Effect of dye concentration on immobilized TP catalyzed dye removal. (pH 2.0, *T* = 24°C, 50 mM of H_2_O_2_ and 0.7 g of immobilized TP for 10 min).

Studies were carried out at different CR concentrations (20–200 mg/L), keeping all other parameters constant. Results are shown in Figure 4. Initial dye concentration present in the aqueous phase has a significant influence on any enzyme-mediated reaction.

If the amount of enzyme concentration is kept constant, the reaction rate increases with increasing CR concentration. A maximum value of approximately 16 mg/L min was attained for CR concentrations above 175 mg/L. After obtaining the equilibrium state any further addition of the substrate did not affect significantly the reaction rate. Analogically, the discoloration yield increases with dye concentration. Maximum removal (80%) was obtained for dye concentrations above 120 mg/L.

### 4.6. Optimum Concentration of H_2_O_2_

**Figure 5:**
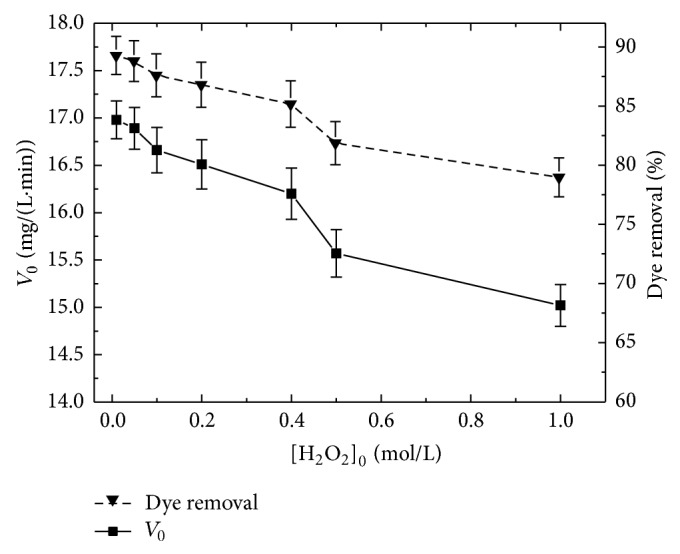
Effect of H_2_O_2_ dose on immobilized TP catalyzed dye removal. (pH 2.0, *T* = 24°C, 180 mg/L of CR and 0.7 g of immobilized TP for 10 min).

Hydrogen peroxide acts as a cosubstrate to activate the enzymatic action of peroxidase radical. It contributes in the catalytic cycle of peroxidase, to oxidize the native enzyme to form an enzymatic intermediate, which accepts the aromatic compound to carry out its oxidation to a free radical form [50]. Experiments were carried out to find out how H_2_O_2_ dose affects the rate and the yield of discoloration. The conversion of dye by varying the H_2_O_2_ dose (0.01–1 M) in the reaction mixture was conducted by keeping all other experimental conditions constant at their optimal values. From Figure 5, the enzyme catalytic capacity was maximal for H_2_O_2_ concentrations below 10 mM under the specified experimental conditions. This result may be due to the fact that inhibition by higher concentrations of H_2_O_2_ may occur especially in a limited particle volume. Partial denaturation of immobilized TP by high H_2_O_2_ concentrations could also be a cause of rate and yield decrease.

### 4.7. Kinetics of CR Discoloration under Optimal Conditions by Immobilized TP

**Figure 6:**
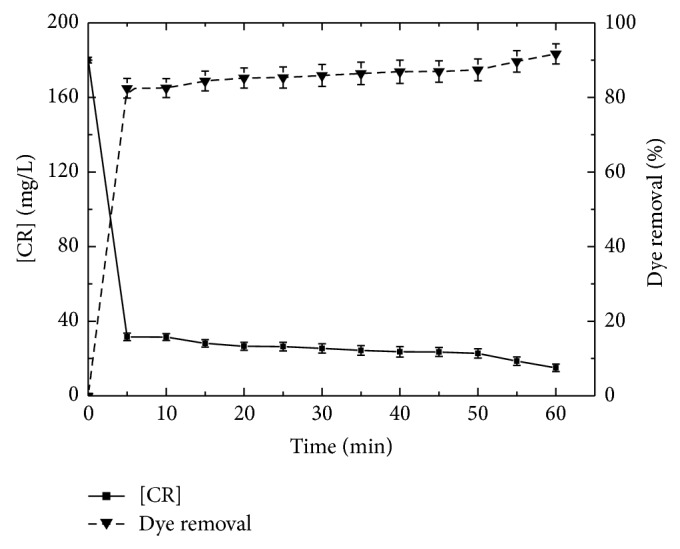
Dye removal pattern with immobilized TP as a function of contact time under optimal conditions (180 mg/L of CR, 10 mM of H_2_O_2_, pH 2.0, *T* = 24°C and 0.7 g of immobilized TP).

Under optimized conditions, 85% of the azo dye was degraded within five minutes and total discoloration of the reaction mixture was achieved after about 1 hour (Figure 6).

### 4.8. Effect of Diffusion on Dye Degradation

Based on (1) the experimental effectiveness factor *η* was determined from the ratio of the biodegradation rate with diffusion limitation (obtained with immobilized TP particles) to the biodegradation rate with no diffusion limitation (obtained with free enzyme), neglecting external resistance which means that the surface concentration is equal to bulk value. The Thiele modulus was calculated from the experimental effectiveness factors using (3) in order to evaluate the intraparticle mass transfer resistance. The effective diffusion coefficient was calculated from the Thiele modulus in (4). All these values were presented in Table 3.

The azo dye diffusivity in the bead was determined adjusting a first order reaction model to the experimental data in order to simplify the mathematical model based on the chemical engineering principles of diffusion and reaction of organic matter in the bead.

The experimental effectiveness factor was smaller than unity. The bioconversion is therefore diffusion limited. From Table 3, the values of Φ found to be nonnegligible showed the influence of intraparticle mass transfer resistance on the overall biodegradation rate. Diffusion coefficient of CR in calcium alginate-immobilized TP beads is calculated. It can be explained that the biopolymer first reduces the volume available for the Congo red to move in; this is termed the exclusion effect. Second, the impenetrable part of biopolymer increases the path length for movement of dye; this is termed the obstruction effect. The predictive value of these concepts is limited, but is useful in visualizing the process.

For small values of Φ, *η* approaches 1.0. Then intraparticle mass transport has no effect on the biodegradation rate per particle; the chemical step controls the rate. For Φ > 5 intraparticle diffusion has a large effect on the biodegradation rate [51]. In our study we can say that bioconversion process is, to a certain extent, affected by diffusional limitations. The determination of diffusional parameters is of great importance in immobilized enzyme bioreactor design and operation [34].

### 4.9. Reusability

**Figure 7:**
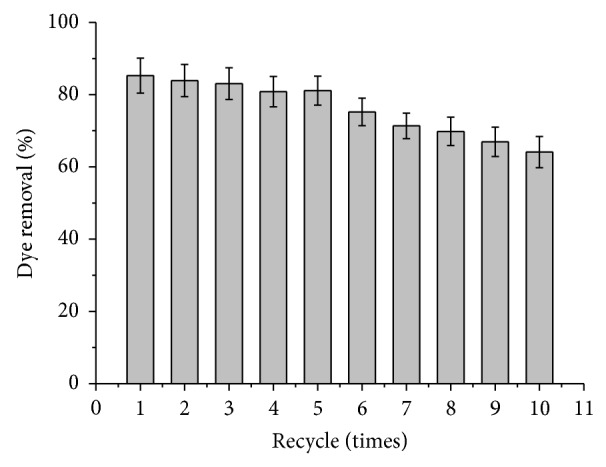
Dye removal pattern at 180 mg/L with repeated application of immobilized TP. (180 mg/L of CR, 10 mM of H_2_O_2_, pH 2.0, *T* = 24°C and 0.7 g of immobilized TP).

The objective of the immobilization is the reusability of the matrix in the process. Therefore investigations were carried out to assess repeated usability of entrapped TP beads for dye removal. The results obtained are shown in Figure 7. The immobilized enzyme could be easily removed and assessed for its remained catalytic activity. To demonstrate the reusability of encapsulated enzyme, capsules were separated after 5 min of reaction time and then rinsed thoroughly with distilled water. The capsules were used for subsequent batches. After five repeated tests, immobilized TP retained 95% of its initial discoloration performance (Figure 7). Other investigators for immobilized TP on the other carrier observed that 50% of the initial activity was lost after five cycles [52, 53].

## 5. Conclusion 

Application of free enzyme in industrial processes is not economically viable in batch or continuous processes. Enzyme immobilization by entrapment is a rational solution in the design at industrial scale. The objective of this study is to obtain the maximum degradation percentage of Congo red by alginate entrapped turnip peroxidase. The preparation and application of immobilized turnip peroxidase in calcium alginate beads for dye removal from aqueous solution was investigated. The experimental results obtained in the present work revealed the effectiveness of the encapsulated peroxidase in dye removal. The performance of Congo red removal was found to be highly dependent on pH, temperature, dye, and hydrogen peroxide concentrations. The encapsulated enzyme activity shows higher relative activity in acidic solutions under ambient temperature. The optimized discoloration reaction is completed within less than half an hour and repeated application of enzyme, up to ten cycles, was observed to be feasible with immobilized enzyme.

The contribution of diffusion resistance in the support particles on the overall process rate was estimated by predicting the effectiveness factor *η*, Thiele modulus Φ, and the effective diffusion coefficient *D*_*e*_. It was also shown that the diffusion resistance has a significant effect on Congo red biodegradation and should not be ignored in any engineering analysis.

## Figures and Tables

**Table 1:** Characteristics of Congo red [39, 40].

Name of the dye	Congo red
CI number	22120
Chemical name	3,3′-[[1,1′-Biphenyl]-4,4′-diylbis-(azo)] bis[4-amino-1-naphthalenesulfonic acid] disodium salt.
Solubility	Soluble in water, ethanol; very slightly soluble in acetone
Molecular formula	C_32_H_22_N_6_O_6_S_2_Na_2_
Molecular Weight	696.67 g/mol
Color change at pH	Blue (3.0) to red (5.0)
Hue	Red in soluble state (λ_max⁡_—500 nm)
Chemical class	Di-azo

**Table 2:** Activities of crude and purified turnip peroxidase TP.

Fraction	Total activity (U)	Total protein (mg)	Specific activity (U/mg)	Recovery (%)	Purification fold
Crude enzyme	62.790	12.722	4.936	100	—
Acetone precipitation	51.975	8.200	6.338	82.78	1.284

**Table 3:** Values of Thiele modulus (Φ), effectiveness factor *η*, and effective diffusion coefficient (*D*_*e*_) for the discoloration of CR by immobilized TP (*T* = 24°C; pH: 2.0 and 0.7 g of immobilized TP).

Parameter	Value
*k* (s^−1^)	0.0014
*η*	0.598
Φ	1.215
*D*_*e*_ (cm^2^/s)	2.7 × 10^−6^

The first-order biodegradation rate constant (*k*) was given from our previous work [29].
